# Positive psychology interventions: a meta-analysis of randomized controlled studies

**DOI:** 10.1186/1471-2458-13-119

**Published:** 2013-02-08

**Authors:** Linda Bolier, Merel Haverman, Gerben J Westerhof, Heleen Riper, Filip Smit, Ernst Bohlmeijer

**Affiliations:** 1 Department of Public Mental Health, Trimbos Institute, Netherlands Institute of Mental Health and Addiction, P.O. Box 725 3500 AS, Utrecht, the Netherlands; 2 Innovation Centre of Mental Health & Technology, Trimbos Institute, Netherlands Institute of Mental Health and Addiction, Utrecht, The Netherlands; 3 Department of Psychology, Health and Technology, University of Twente, Enschede, The Netherlands; 4 The EMGO institute for Health and Care research, VU University, Amsterdam, The Netherlands; 5 Innovation Incubator, Leuphana University, Lueneburg, Germany; 6 Department of Epidemiology and Biostatistics, VU University Medical Centre, Amsterdam, The Netherlands

**Keywords:** Well-being, Depression, Positive psychology, Interventions, Effectiveness, Randomized controlled trials, Meta-analysis

## Abstract

**Background:**

The use of positive psychological interventions may be considered as a complementary strategy in mental health promotion and treatment. The present article constitutes a meta-analytical study of the effectiveness of positive psychology interventions for the general public and for individuals with specific psychosocial problems.

**Methods:**

We conducted a systematic literature search using PubMed, PsychInfo, the Cochrane register, and manual searches. Forty articles, describing 39 studies, totaling 6,139 participants, met the criteria for inclusion. The outcome measures used were subjective well-being, psychological well-being and depression. Positive psychology interventions included self-help interventions, group training and individual therapy.

**Results:**

The standardized mean difference was 0.34 for subjective well-being, 0.20 for psychological well-being and 0.23 for depression indicating small effects for positive psychology interventions. At follow-up from three to six months, effect sizes are small, but still significant for subjective well-being and psychological well-being, indicating that effects are fairly sustainable. Heterogeneity was rather high, due to the wide diversity of the studies included. Several variables moderated the impact on depression: Interventions were more effective if they were of longer duration, if recruitment was conducted via referral or hospital, if interventions were delivered to people with certain psychosocial problems and on an individual basis, and if the study design was of low quality. Moreover, indications for publication bias were found, and the quality of the studies varied considerably.

**Conclusions:**

The results of this meta-analysis show that positive psychology interventions can be effective in the enhancement of subjective well-being and psychological well-being, as well as in helping to reduce depressive symptoms. Additional high-quality peer-reviewed studies in diverse (clinical) populations are needed to strengthen the evidence-base for positive psychology interventions.

## Background

Over the past few decades, many psychological treatments have been developed for common mental problems and disorders such as depression and anxiety. Effectiveness has been established for cognitive behavioral therapy [1,2], problem-solving therapy [3] and interpersonal therapy [4]. Preventive and early interventions, such as the Coping with Depression course [5], the Don’t Panic course [6] and Living Life to the Full [7,8] are also available. The existing evidence shows that the mental health care system has traditionally focused more on treatment of mental disorders than on prevention. However, it is recognized that mental health is more than just the absence of mental illness, as expressed in the World Health Organization’s definition of mental health:

*Mental health is a state of well-being in which the individual realizes his or her own abilities, can cope with the normal stresses of life, can work productively, and is able to make a contribution to his or her community*[9].

Under this definition well-being and positive functioning are core elements of mental health. It underscores that people can be free of mental illness and at the same time be unhappy and exhibit a high level of dysfunction in daily life [10]. Likewise, people with mental disorders, can be happy by coping well with their illness and enjoy a satisfactory quality of life [11]. Subjective well-being refers to a cognitive and/or affective appraisal of one’s own life as a whole [12]. Psychological well-being focuses on the optimal functioning of the individual and includes concepts such as mastery, hope and purpose in life [13,14]. The benefits of well-being are recorded both in cross-sectional and longitudinal research and include improved productivity at work, having more meaningful relationships and less health care uptake [15,16]. Well-being is also positively associated with better physical health [17-19]. It is possible that this association is mediated by a healthy lifestyle and a healthier immune system, which buffers the adverse influence of stress [20]. In addition, the available evidence suggests that well-being reduces the risk of developing mental symptoms and disorders [21,22] and helps reduce mortality risks in people with physical disease [23].

Seligman and Csikszentmihaly’s (2000) pioneered these principles of positive psychology in their well-known article entitled ‘Positive psychology: An introduction’, published in a special issue of the *American Psychologist.* They argued that a negative bias prevailed in psychology research, where the main focus was on negative emotions and treating mental health problems and disorders [24]. Although the basic concepts of well-being, happiness and human flourishing have been studied for some decades [12,25-27], there was a lack of evidence-based interventions [24]. Since the publication of Seligman and Csikszentmihaly’s seminal article, the positive psychology movement has grown rapidly. The ever-expanding International Positive Psychology Association is among the most extensive research networks in the world [28] and many clinicians and coaches embrace the body of thought that positive psychology has to offer.

Consequently, the number of evaluation studies has greatly increased over the past decade. Many of these studies demonstrated the efficacy of positive psychology interventions such as counting your blessings [29,30], practicing kindness [31], setting personal goals [32,33], expressing gratitude [30,34] and using personal strengths [30] to enhance well-being, and, in some cases, to alleviate depressive symptoms [30]. Many of these interventions are delivered in a self-help format. Sin and Lyubomirsky (2009) conducted a meta-analytical review of the evidence for the effectiveness of positive psychology interventions (PPIs). Their results show that PPIs can indeed be effective in enhancing well-being (r = 0.29, standardized mean difference Cohen’s *d* = 0.61) and help to reduce depressive symptom levels in clinical populations (r = 0.31, Cohen’s *d* = 0.65). However, this meta-analysis had some important limitations. First, the meta-analysis included both randomized studies and quasi-experimental studies. Second, study quality was not addressed as a potential effect moderator. In recent meta-analyses, it has been shown that the treatment effects of psychotherapy have been overestimated in lower quality studies [35,36]. The lack of clarity in the inclusion criteria constitutes a third limitation. Intervention studies, although related to positive psychology but not strictly developed within this new framework (e.g. mindfulness, life-review) were included in the meta-analysis. However, inclusion of these studies reduces the robustness of the results for pure positive psychology interventions.

### Present study

The aim of the present study is to conduct a meta-analysis of the effects of specific positive psychology interventions in the general public and in people with specific psychosocial problems. Subjective well-being, psychological well-being and depressive symptoms were the outcome measures. Potential variables moderating the effectiveness of the interventions, such as intervention type, duration and quality of the research design, were also examined. This study will add to the existing literature and the above meta-analytical review [37] by 1) only including randomized controlled studies, 2) taking the methodological quality of the primary studies into account, 3) including the most recent studies (2009 – 2012), 4) analyzing not only post-test effects but also long-term effects at follow up, and 5) applying clear inclusion criteria for the type of interventions and study design.

## Method

### Search strategy

A systematic literature search was carried out in PsychInfo, PubMed and the Cochrane Central Register of Controlled Trials, covering the period from 1998 (the start of the positive psychology movement) to November 2012. The search strategy was based on two key components: there should be a) a specific positive psychology intervention, and b) an outcome evaluation. The following MeSH terms and text words were used: “well-being” or “happiness” or “happy*”, “optimism”, “positive psychology” in combination with “intervention”, “treatment”, “therapy” and “prevention”. This was combined with terms related to outcome research: “effect*”, or “effic*”, or “outcome*”, or “evaluat*”. We also cross-checked the references from the studies retrieved, the earlier meta-analysis of Sin & Lyubomirsky (2009) and two other reviews of positive psychological interventions [38,39]. The search was restricted to peer-reviewed studies in the English language.

### Selection of studies

Two reviewers (LB and MH) independently selected potentially eligible studies in two phases. At the first phase, selection was based on title and abstract, and at the second phase on the full-text article. All studies identified as potentially eligible by at least one of the reviewers during the first selection phase, were re-assessed at the second selection phase. During the second phase, disagreements between the reviewers were resolved by consensus. The inter-rater reliability (kappa) was 0.90.

The inclusion criteria were as follows:

• Examination of the effects of a positive psychology intervention. A positive psychology intervention (PPI) was defined in accordance with Sin and Lyubomirsky’s (2009) article as a psychological intervention (training, exercise, therapy) primarily aimed at raising positive feelings, positive cognitions or positive behavior as opposed to interventions aiming to reduce symptoms, problems or disorders. The intervention should have been explicitly developed in line with the theoretical tradition of positive psychology (usually reported in the introduction section of an article).

• Randomization of the study subjects (randomizing individuals, not groups) and the presence of a comparator condition (no intervention, placebo, care as usual).

• Publication in a peer-reviewed journal.

• At least one of the following are measured as outcomes: well-being (subjective well-being and/or psychological well-being) or depression (diagnosis or symptoms).

• Sufficient statistics are reported to enable the calculation of standardized effect sizes.

If necessary, authors were contacted for supplementary data. We excluded studies that involved physical exercises aimed at the improvement of well-being, as well as mindfulness or meditation interventions, forgiveness therapy, life-review and reminiscence interventions. Furthermore, well-being interventions in diseased populations not explicitly grounded in positive psychology theory (‘coping with disease courses’) were excluded. Apart from being beyond the scope of this meta-analysis, extensive meta-analyses have already been published for these types of intervention [40-42]. This does not imply that these interventions do not have positive effects on well-being, a point which will be elaborated on in the discussion section of this paper.

### Data extraction

Data extraction and study quality assessment were performed by one reviewer (LB) and independently checked by a second reviewer (MH). Disagreements were resolved by consensus. Data were collected on design, intervention characteristics, target group, recruitment methods, delivery mode, number of sessions, attrition rates, control group, outcome measures and effect sizes (post-test and at follow up of at least 3 months). The primary outcomes in our meta-analysis were subjective well-being (SWB), psychological well-being (PWB) and depressive symptoms/depression.

The methodological quality of the included studies was assessed using a short scale of six criteria tailored to those studies and based on criteria established by the Cochrane collaboration [43]: 1) Adequacy of randomization concealment, 2) Blinding of subjects to the condition (blinding of assessors was not applicable in most cases), 3) Baseline comparability: were study groups comparable at the beginning of the study and was this explicitly assessed? (Or were adjustments made to correct for baseline imbalance using appropriate covariates), 4) Power analysis: is there an adequate power analysis and/or are there at least 50 participants in the analysis?, 5) Completeness of follow up data: clear attrition analysis and loss to follow up < 50%, 6) Handling of missing data: the use of intention-to-treat analysis (as opposed to a completers-only analysis). Each criterion was rated as 0 (study does not meet criterion) or 1 (study meets criterion). The inter-rater reliability (kappa) was 0.91. The quality of a study was assessed as high when five or six criteria were met, medium when three or four criteria were met, and low when zero, one or two criteria were met. Along with a summary score, the aspects relating to quality were also considered individually, as results based on composite quality scales can be equivocal [44]. Table 1 shows the quality assessment for each study. The quality of the studies was scored from 1 to 5 (M = 2.56; SD = 1.25). Twenty studies were rated as low, 18 were of medium quality and one study was of high quality. None of the studies met all quality criteria. The average number of participants in the analysis was rather high (17 out of 39 studies scored positive on this criterion), although none of the studies reported an adequate power analysis. Also, baseline comparability was frequently reported (26/39 studies). On the other hand, independence in the randomization procedure was seldom reported (7/39 studies) and an intention-to-treat analysis was rarely conducted (3/39 studies).

**Table 1:** Quality assessment per study

**Study**	**1**	**2**	**3**	**4**	**5**	**6**	**Total**
Abbott 2009 [78]	0	0	1	1	0	1	3
Boehm 2011 [73]	0	1	1	1	0	1	4
Buchanan 2010 [56]	0	0	0	1	0	0	1
Burton 2004 [64]	0	1	1	1	1	0	4
Cheavens 2006 [76]	0	0	1	0	1	0	2
Emmons 2006 study 1	0	1	0	1	0	0	2
Emmons 2006 study 3	0	1	0	1	0	0	2
Fava 1998 [82]	0	1	0	0	1	0	2
Fava 2005 [83]	0	1	0	0	0	0	1
Feldman 2012 [60]	0	0	0	1	0	0	1
Frieswijk 2005	0	0	0	1	0	0	1
Gander 2012 [74]	0	1	1	1	0	0	3
Goldstein 2007 [84]	0	0	0	1	0	0	1
Grant 2009 [79]	0	0	0	0	0	0	0
Grant 2012 [82]	1	1	1	1	0	0	4
Green 2006 [33]	0	0	0	1	1	0	2
Hurley 2012 [61]	0	0	1	1	0	0	2
King 2001 [66]	0	1	0	0	1	0	2
Kremers 2006 [57]	0	0	1	1	1	0	3
Layous 2012 [75]	0	1	1	1	0	0	3
Lichter 1980 study 2 [80]	0	0	0	0	1	0	1
Luthans 2008 [65]	1	1	1	1	0	0	4
Luthans 2010 study 1 [72]	0	1	1	1	1	0	4
Lyubomirsky 2006 study 2 [58]	0	1	1	1	1	0	4
Lyubomirsky 2011 [67]	0	1	1	1	0	0	3
Martinez 2010 [68]	0	1	1	1	1	0	4
Mitchell 2009 [69]	1	1	1	1	0	1	5
Page 2012 [62]	1	0	1	0	0	0	2
Peters 2010 [70]	0	1	1	1	1	0	4
Quoidbach 2009 [59]	1	0	1	0	0	0	2
Schueller 2012 [63]	1	0	1	1	0	0	3
Seligman 2005 [30]	0	1	1	1	1	0	4
Seligman 2006 study 1 [51]	0	0	1	0	0	0	1
Seligman 2006 study 2 [51]	0	1	1	0	1	0	3
Shapira 2010 [55]; Mongrain 2011 [53]; Sergeant 2011 [54]; Mongrain 2012 [52]	1	1	1	1	0	0	4
Sheldon 2002 [32]	0	1	1	1	1	0	4
Sheldon 2006 [34]	0	1	1	0	0	0	2
Spence 2007 [81]	0	0	1	0	0	0	1
Wing 2006 [71]	0	1	0	1	0	0	2
Total	7	23	26	27	14	3	100

Index:

1 = Randomization concealment.

2 = Blinding of subjects.

3 = Baseline comparability.

4 = Power analysis or N>=50.

5 = Completeness of follow up data.

6 = Intention-to-treat analysis.

### Meta-analysis

In a meta-analysis, the effects found in the primary studies are converted into a standardized effect size, which is no longer placed on the original measurement scale, and can therefore be compared with measures from other scales. For each study, we calculated effect sizes (Cohen’s *d*) by subtracting the average score of the experimental group (Me) from the average score of the control group (Mc), and dividing the result by the pooled standard deviations of both groups. This was done at post-test because randomization usually results in comparable groups across conditions at baseline. However, if baseline differences on outcome variables did exist despite the randomization, d’s were calculated on the basis of pre- post-test differences: by calculating the standardized pre- post change score for the experimental group (de) and the control group (dc) and subsequently calculating their difference as Δd= de – dc. For example, an effect size of 0.5 indicates that the mean of the experimental group is half a standard unit (standard deviation) larger than the mean of the control group. From a clinical perspective, effect sizes of 0.56 – 1.2 can be interpreted as large, while effect sizes of 0.33 – 0.55 are of medium size, and effects of 0 – 0.32 are small [45].

In the calculation of effect sizes for depression, we used instruments that explicitly measure depression (e.g. the Beck Depression Inventory, or the Center for Epidemiological Studies Depression Scale). For subjective and psychological well-being, we also used instruments related to the construct of well-being (such as positive affect for SWB and hope for PWB). If more than one measure was used for SWB, PWB or depression, the mean of the effect sizes was calculated, so that each study outcome had one effect size. If more than one experimental group was compared with a control condition in a particular study, the number of subjects in the control groups was evenly divided across the experimental groups so that each subject was used only once in the meta-analysis.

To calculate pooled mean effect sizes, we used Comprehensive Meta-Analysis (CMA, Version 2.2.064). Due to the diversity of studies and populations, a common effect size was not assumed and we expected considerable heterogeneity. Therefore, it was decided *a priori* to use the ‘random effects model’. Effect sizes may differ under this model, not only because of random error within studies (as in the fixed effects model), but also as a result of true variation in effect sizes between studies. The outcomes of the random effects model are conservative in that their 95% Confidence Intervals (CIs) are often broad, thus reducing the likelihood of type-II errors.

We tested for the presence of heterogeneity with two indicators. First, we calculated the Q-statistic. A significant Q rejects the null-hypothesis of homogeneity and indicates that the true effect size probably does vary from study to study. Second, the I^2^-statistic was calculated. This is a percentage indicating the study-to-study dispersion due to real differences, over and above random sampling error. A value of 0% indicates an absence of dispersion, and larger values show increasing levels of heterogeneity where 25% can be considered as low, 50% as moderate and 75% as a high level of heterogeneity [46].

Owing to the expected high level of heterogeneity, all studies were taken into account. Outliers were considered, but not automatically removed from the meta-analysis. The procedure of removing outliers which are outside the confidence interval of the pooled effect size is advised when a common effect size is assumed. However, in our meta-analysis, high dispersion was expected and therefore only the exclusion of Cohen’s *d* > 2.5 from the final sample was planned.

Subgroup analyses were performed by testing differences in Cohen’s *d’s* between subgroups. Six potential moderators were determined based on previous research and the characteristics of the investigated interventions and studies: 1) Self-selected sample/not self-selected: did the participants know that the aim of the intervention was to make them feel better?; 2) Duration: less than four weeks, four to eight weeks, or more than eight weeks; 3) Type of intervention: self-help, group intervention, or individual therapy; 4) Recruitment method: community (in a community center, local newspapers), internet, by referral/hospital, at university; 5) Psychosocial problems (Yes/none): was the data based on a group with certain psychosocial problems or was the study open to everyone?; 6) Quality rating: low (score 1 or 2), medium (score 3 or 4) or high (score 5 or 6). The impact of the duration and quality ratings was also assessed using meta-regression.

Results of meta-analysis may be biased due to the fact that studies with non-significant or negative results are less likely to be published in peer-reviewed journals [47]. In order to address this issue, we used three indices: funnel plots, the Orwin’s fail-safe number and the Trim and Fill method. A funnel plot is a graph of effect size against study size. When publication bias is absent, the observed studies are expected to be distributed symmetrically around the pooled effect size. The Orwin’s fail-safe number indicates the number of non-significant unpublished studies needed to reduce the overall significant effect to non-significance (according to a self-stated criterium) [48]. The effect size can be considered to be robust if the number of studies required to reduce the overall effect size to a non-significant level exceeds 5 K + 10, where K is the number of studies included. If asymmetry is found in the funnel plot, the Trim and Fill method adjusts the pooled effect size for the outcomes of missing studies [49]. Imputing missing studies restores the symmetry in the funnel plot and an adjusted effect size can be calculated.

For the reporting of the results of this meta-analysis, we applied Preferred Reporting Items for Systematic Reviews and Meta-Analyses (PRISMA) guidelines [50].

## Results

### Description of studies

**Figure 1:**
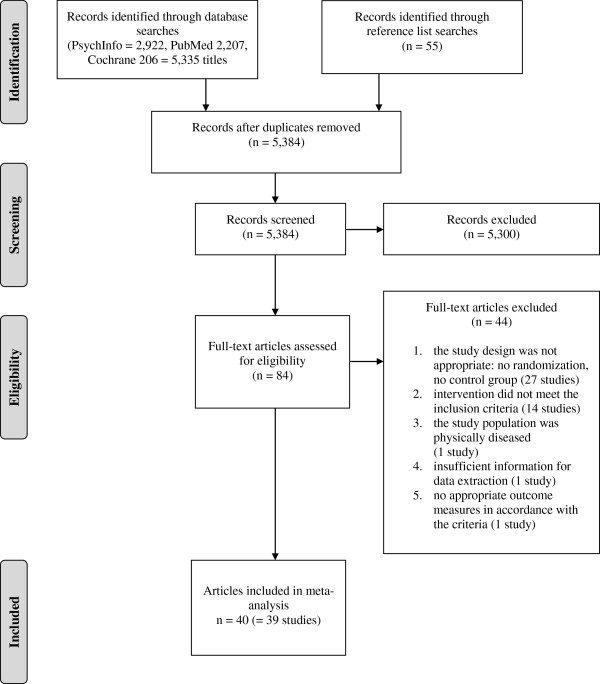
Flow diagram.

The selection process is illustrated in Figure 1. First, 5,335 titles were retrieved from databases and 55 titles were identified through searching the reference list accompanying the meta-analysis by Sin and Lyubomirsky (2009) [37] as well as two other literature reviews of positive psychological interventions [38,39]. After reviewing the titles and abstracts and removing duplicates, 84 articles were identified as being potentially eligible for inclusion in our study. Of these 84 articles, 40 articles in which 39 studies were described, met our inclusion criteria (of these, 17 articles describing 19 studies were also included in the meta-analysis by Sin and Lyubomirsky, 2009). In two articles [29,51] two studies were described, and one study [52-55] was published in four articles.

The characteristics of the studies included are described in Table 2. The studies evaluated 6,139 subjects, 4,043 in PPI groups and 2,096 in control groups. Ten studies compared a PPI with a no-intervention control group [29,51,56-63], 17 studies compared a PPI with a placebo intervention [29,30,32,34,52-55,64-75], seven studies with a waiting list control group [33,76-81] and five studies with another active intervention (care as usual) [51,82-85]. A minority of seven studies [51,57,76,77,82,83] applied inclusion criteria to target a specific group with psychosocial problems such as depression and anxiety symptoms. Half of the studies, 19 in total, recruited the subjects (not necessarily students) through university [29,32,34,51,56,58-61,64-68,70,72,75,80,85]. In seven studies subjects were recruited in the community [33,57,71,73,76,77,81], in four studies by referral from a practitioner or hospital [29,51,82,83], in three studies in an organization [62,78,79] and six studies recruited through the internet [30,52-55,63,69,74,84]. Twenty-eight studies measured subjective well-being, 20 studied psychological well-being and 14 studied depressive symptoms. Half of the studies (20) were aimed at adult populations [29,30,33,51-56,62,63,65,69,71,73,74,76,78,79,81-84]. A substantial number of studies (17) were aimed at college students [29,32,34,51,58-61,64,66-68,70,72,75,80,85] and two studies were aimed at older subjects [57,77]. In most studies (26) the PPI was delivered in the form of self-help [29,30,34,52-56,58,59,61,63-71,73-75,77,78,80,84,85]. Eight studies used group PPIs [32,33,51,57,60,62,72,76] and five used individual PPIs [51,79,81-83]. Intensity varied considerably across studies, ranging from a short one-day exercise [70] and a two-week self-help intervention [65] to intensive therapy [51,82,83] and coaching [33,81].

**Table 2:** Characteristics of randomized controlled trials examining the effects of positive psychology interventions

**Author**	**Intervention**	**Session (number), duration**	**Mean age (range or SD)**	**Delivery**	**Recruitment**	**Self-selection**	**Psychosocial problems / inclusion criteria**	**Control Group**	**N analyzed (post test)**	**Attrition rate, % (post test)**	**Outcome measures**	**Follow-up (min. 3 months)**
Abbott 2009 [78]	ResilienceOnline	7, 10w	43	Self-help	Organization	Self-selected	None	Waiting list	Ne=26 Nc=27	41.5%	PWB: AHI DEP: DASS-21	-
Boehm 2011 [73]	Optimism and gratitude exercise	6, 6w	35.6 (11.4)	Self-help	Community	Self-selected	None	Placebo	Ne=146 Nc=74	?	SWB: SWLS	-
Buchanan 2010 [56]	Doing acts of kindness	10, 10d	26 (18–60)	Self-help	University	Self-selected	None	No intervention	Ne=28 Nc=28	0% (? nr)	SWB: SWLS	-
Burton 2004 [64]	Writing about positive experiences	3, 3d	College-based sample 18.6 (0.95)	Self-help	University	Not self-selected	None	Placebo	Ne=48 Nc=42	0%	SWB: PA	-
Cheavens 2006 [76]	Hope therapy	8, 8w	49 (32–64)	Group	Community	Self-selected	Inclusion criteria unclear	Waiting list	Ne=17 Nc=15	12% 22% T: 18%	PWB: SHS DEP: CES-D	-
Emmons 2006 study 1 [29]	Practising gratitude by counting one’s blessings	10, 10w	U (students)	Self-help	University	Not self-selected	None	Placebo	Ne=65 Nc=67	T: 4%	SWB: Life as a whole, upcoming week, PA	-
Emmons 2006 study 3 [29]	Practising gratitude by counting one’s blessings	21, 3w	49 (22–77)	Self-help	Referral/hospital	Not self-selected	None	No intervention	Ne=33 Nc=32	0% (? nr)	SWB: Life as a whole, upcoming week, PA (self-report and observed)	-
Fava 1998 [82]	Well-being therapy	8, 16w	28.4 (6.5)	Individual	Referral/hospital	Self-selected	Diagnosis of MDD or AD, succesful response to treatment	TAU	Ne=10 Nc=10	0%	PWB: RPWB DEP: CID, SQ subscale	-
Fava 2005 [83]	Well-being therapy	8, 16w	41.9 (12)	Individual	Referral/hospital	Self-selected	Diagnosis of GAD	TAU	Ne=8 Nc=8	20%	PWB: RPWB DEP: CID, SQ subscale	1 yr (not in study)
Feldman 2012 [60]	Hopeful goal-directed thinking	1, 1d	18.7 (18–22)	Group	University	Not self-selected	None	No intervention	Ne=37 Nc=29	24.7%	PWB: GSHS, PIL	
Frieswijk 2005 [77]	Self-management positive bibliotherapy	5, 10w	72.9 (6.2)	Self-help	Community	Self-selected	Slightly or moderately frail (>=65 GFI)	Waiting list	Ne=79 Nc=86	18.4% 10.4% T: 14.5%	SWB: SPF-IL PWB: MS	6 m
Gander 2012 [74]	9 exercises: gratitude visit three good things (1 and 2 weeks), strengths, three funny things, social exercises	7, 1w 14, 2w	44.9 (10.07)	Self-help	Internet, magazine	Self-selected	None	Placebo	Ne=559 Nc=63	74%	PWB: AHI DEP: CES-D	6 m
Goldstein 2007 [84]	Cultivating sacred moments	15, 3w	(22–44)	Self-help	Internet	Self-selected	None	TAU	Ne=35 Nc=38	14.6% 9.5% T: 12.0%	SWB: SWLS PWB: RPWB	-
Grant 2009 [79]	Executive coaching	6, 8-10w	49.8	Group and individual	Organization	Self-selected	None	Waiting list	Ne=21 Nc=20	18%	DEP: DASS-21	-
Grant 2012 [85]	Solution-focused coaching	1, 1d	20.5 (5.4)	Self-help	University	Self-selected	None	TAU	Ne=117 Nc=108	0% (? nr)	SWB: PA	-
Green 2006 [33]	Life coaching and attainment of goals	10, 10w	42.7 (18–60)	Group	Community	Self-selected	None	Waiting list	Ne=25 Nc=25	10.7% 10.7% T: 10.7%	SWB: SWLS, PA PWB: RPWB, SHS	-
Hurley 2012 [61]	Savoring the moment	14, 2w	19.5 (2.06)	Group / Self-help	University	Not self-selected	None	No intervention	Ne=94 Nc=99	37.7% 39.6% T: 38.7%	SWB: PA DEP: BDI	-
King 2001 [66]	Writing about best possible selves	4, 4d	21 (18–42)	Self-help	University	Not self-selected	None	Placebo	Ne=19 Nc=16	0%	SWB: PA	-
Kremers 2006 [57]	Self-management positive group course	6, 6w	64.3 (7)	Group	Community	Self-selected	Single and lonely	No intervention	Ne=46 Nc=73	17.0% 7.6% T: 16.2%	SWB: SPF-IL	6 m
Layous 2012 [75]	Best possible selves exercise	4, 4w	19.1 (1.8)	Self-help Group	University	Not self-selected	None	Placebo	Ne=80 Nc=37	?	SWB: PA PWB: NS	-
Lichter 1980 study 2 [80]	Rehearsal of positive statements	14, 2w	U (students)	Individual	University	Not self-selected	None	Waiting list	Ne=25 Nc=23	0% (? nr)	SWB: AF1 DEP: BDI	-
Luthans 2008 [65]	Online well-being program (PsyCap)	2, 2w	32.2	Self-help	University	Self-selected	None	Placebo	Ne=187 Nc=177	6.0% 4.8% T: 5.5%	PWB: PCQ	-
Luthans 2010 study 1 [72]	PsyCap training	1, 2 h	21.1 (2.66)	Group	University	Not self-selected	None	Placebo	Ne=153 Nc=89)	0%	PCQ	-
Lyubomirsky 2006 study 2 [58]	Thinking about positive life experiences	3, 3d	19.5 (2.6)	Self-help	University	Not self-selected	None	No intervention	Ne=26 Nc=36	0%	SWB: SWLS, PA	-
Lyubomirsky 2011 [67]	Expressing optimism or gratitude	8, 8w	19.7 (18–46)	Self-help	University	Self-selected	None	Placebo	Ne=218 Nc=101	T: 10.1%	SWB: PLA, SWLS, SHS(2)	6 m
Martinez 2010 [68]	Practising gratitude by counting one’s blessings	14, 2w	20.7 (1.5)	Self-help	University	Not self-selected	None	Placebo	Ne=41 Nc=34	34.0%	SWB: PA, GA (self-report and observed)	-
Mitchell 2009 [69]	Online intervention Use your strenghts in a new way	3, 3w	37 (18–62)	Self-help	Internet	Self-selected	None	Placebo	Ne=48 Nc=54	64.6% 57.4% T: 60.8%	SWB: PWI-A, SWLS, PA PWB: OTH DEP: DASS-21	3 m
Page 2012 [62]	Working for Wellness Program	6, 6w	39.7 (10.0)	Group	Organization	Self-selected	None	No intervention	Ne=13 Nc=10	58.1% 66.7% T: 62.3%	SWB: SWLS, PA PWB: SPWB	6 month
Peters 2010 [70]	Positive future thinking	1, 1d	29.7 (21–50)	Self-help	University	Not self-selected	None	Placebo	Ne=44 Nc=38	0%	SWB: PA	-
Quoidbach 2009 [59]	Projecting a positive self in the future	14, 2w	32.5	Self-help	University	Not self-selected	None	No intervention	Ne=15 Nc=57	T: 49.5%	SWB: SHS(2)	-
Schueller 2012 [63]	Package of 2, 4 or 6 positive psychology exercises (active-constructive responding, gratitude visit, life summary, three good things, savoring, strengths)	14, 2w 28, 4w 42, 6w	42.4 (12.1)	Self-help	Internet	Self-selected	None	No intervention	Ne=457 Nc=204	54.7% 42.5% T: 55.4%	DEP: CES-D	-
Seligman 2005 [30]	Strenghts excercises (2), gratitude (1), positive thinking (2)	7, 1w	64% between5-54	Self-help	Internet	Self-selected	None	Placebo	Ne=341 Nc=70	T: 28.8%	PWB: SHI DEP: CES-D	6 m
Seligman 2006 study 1 [51]	Group positive psychotherapy	6, 6w	U (students)	Group	University	Self-selected	Mild to moderate depressive symptoms (BDI 10–24)	No intervention	Ne=14 Nc=20	26.3% 4.8% T: 15.0%	SWB: SWLS DEP: BDI	3, 6 (in study), 12 m
Seligman 2006 study 2 [51]	Individual positive psychotherapy	14, 12w	U (adults)	Individual	Referral/hospital	Self-selected	Clinical diagnosis of MDD	TAU	Ne=11 Nc=9	15.4% 40.0% T: 28.6%	SWB: SWLS PWB: PPTI DEP: HRSD, ZSRS	-
Shapira 2010 [55]; Mongrain 2011 [53]; Sergeant 2011 [54]; Mongrain 2012 [52]	Three good things, signature strengths, self-compassion, optimism, compassionate action, gratitude intervention	7, 1w	34 (11.8)	Self-help	Internet	Self-selected	None	Placebo	Ne=804 Nc=138	75%	PWB: SHI DEP: CES-D	3 (in study), 6 m
Sheldon 2002 [32]	Goal-training program	2, 2w	U (students)	Group	University	Not self-selected	None	Placebo	Ne=36 Nc=42	T: 13.3%	SWB: PA PWB: RPWB	-
Sheldon 2006 [34]	Gratitude or visualizing positive self	14, 2w	U (students)	Self-help	University	Not self-selected	None	Placebo	Ne=44 Nc=24	T: 6.0%	SWB: PA	-
Spence 2007 [81]	Life coaching and attainment of goals	10, 10w	38.6	Individual	Community	Self-selected	None	Waiting list	Ne=20 Nc=17	4.8%15.0% T: 9.8%	SWB: SWLS, PA PWB: RPWB	-
Wing 2006 [71]	Positive writing	3, 3d	40.3 (18–79)	Self-help	Community	Self-selected	None	Placebo	Ne=58 Nc=55	6.3%	SWB: SWLS	-

Abbreviations. U = Unknown; Ne = Number of subjects in experimental group; Nc = Number of subjects in control group; T = Total; nr = Not reported; MDD = Major Depressive Disorder; AD = Anxiety Disorder; GAD = Generalized Anxiety Disorder; AHI = Authentic Happiness Inventory; EASQ = Expanded Attributional Style Questionnaire; GFI = Groningen Frailty Indicator; SWLS = Satisfaction with Life Scales; PA = Positive Affect; SHS = State Hope Scale; PIL = Purpose in Life Test; CES-D = Center for Epidemiological Studies Depression Scale; SQ = Kellner’s Symptom Questionnaire; CID = Clinical Interview for Depression; RPWB = Ryff’s Scales of Psychological Well-being; MHC-SF = Mental Health Continuum-Short Form; HS = Hope Scale; BDI = Beck Depression Inventory; SPF-IL = Social Production Function-Index Level Scale; MS = Mastery Scale; AF-1 = Affectometer 1; PCQ = PsyCap Questionnaire; PLA = Pleasant Affect; SHS(2) = Subjective Happiness Scale; GA = Global Appraisals of subjective well-being; PWI-A = Personal Well-being Index for Adults; OTH = Orientations To Happiness; DASS-21 = Depression, Anxiety and Stress Scales; WB6 = 6 well-being questions; SHI = Steen Happiness Index; PPTI = Positive Psychotherapy Inventory; HRSD = Hamilton Rating Scale for Depression; ZSRS = Zung Self-Rating Scale; LSI-A = Life Satisfaction Index-A; GDS = Geriatric Depression Scale; GSHS = Goal-Specific Hope Scale; NS = Needs Satisfaction.

### Post-test effects

**Figure 2:**
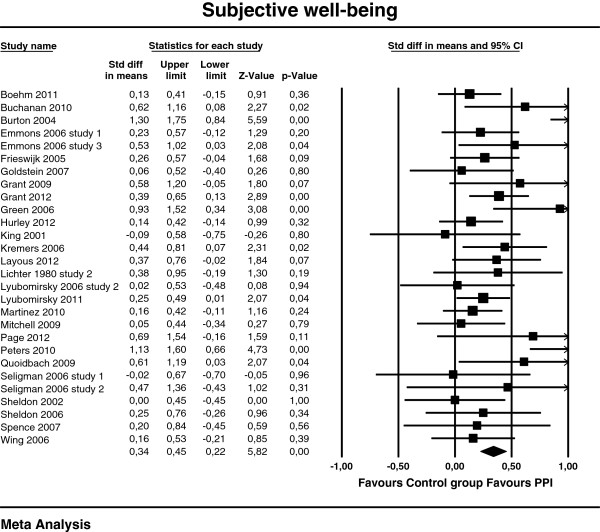
**Post-test effects of positive psychology interventions on subjective well-being. **The square boxes show effect size and sample size (the larger the box, the larger the sample size) in each study, and the line the 95% confidence interval. The diamond reflects the pooled effect size and the width of the 95% confidence interval.

**Figure 3:**
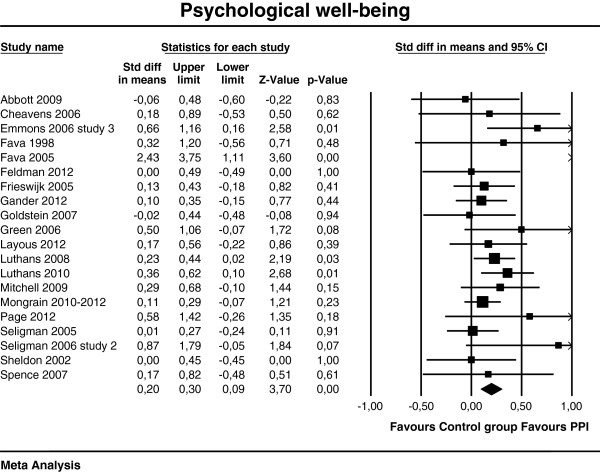
**Post-test effects of positive psychology interventions on psychological well-being. **The square boxes show effect size and sample size (the larger the box, the larger the sample size) in each study, and the line the 95% confidence interval. The diamond reflects the pooled effect size and the width of the 95% confidence interval.

**Figure 4:**
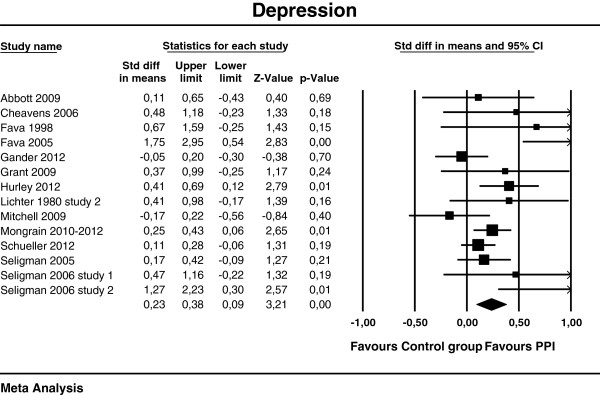
**Post-test effects of positive psychology interventions on depressive symptoms. **The square boxes show effect size and sample size (the larger the box, the larger the sample size) in each study, and the line the 95% confidence interval. The diamond reflects the pooled effect size and the width of the 95% confidence interval.

The random effect model showed that the PPIs were effective for all three outcomes. Results are presented in Table 3. The effect sizes of the individual studies at post-test are plotted in Figures 2, 3 and 4.

**Table 3:** Main effects

**Outcome measures**	**n**	**N**	**Studies**	**Cohen’s *d *(95% CI)**	**Heterogeneity**	**Test for overall effect**
Post-test						
Subjective well-being	28	Ne=1449 Nc=1265	[29,32-34,51,56-59,61,62,64,66-71,73,75,77,79-81,84,85]	0.34 (0.22 – 0.45)	Q=53.5, df=27, T^2^=0.04 (p<.01); I^2^=49.5%	Z=5.82 (p<.01)
Psychological well-being	20	Ne=2511 Nc=977	[29,30,32,33,51-55,60,62,65,69,74-77,81-84]	0.20 (0.09 – 0.30)	Q=26.8, df=19, T^2^=0.01 (p=0.11); I^2^=29.0%	Z=3.65 (p<.01)
Depression	14	Ne=2435 Nc=760	[30,51-55,61,63,69,74,76,80,82,83]	0.23 (0.09 – 0.38)	Q=24.5, df=13, T^2^=0.03 (p=0.03); I^2^=47.0%	Z=3.21 (p<.01)
Follow-up						
Subjective well-being	6	Ne=329 Nc=298	[51,57,62,67,69,77]	0.22 (0.05 – 0.38)	Q=5.05, df=5, T^2^=0.00 (p=0.41); I^2^=1.1%	Z=2.61 (p<.01)
Psychological well-being	6	Ne=1830 Nc=417	[30,52-55,62,69,74,77]	0.16 (0.02 – 0.30)	Q=6.8, df=5, T^2^=0.01 (p=0.24); I^2^=26.0%	Z=2.20 (p=.03)
Depression	5	Ne=1765 Nc=343	[30,51-55,69,74]	0.17 (−0.06 – 0.39)	Q=11.1, df=4, T^2^=0.04 (p=0.03); I^2^=63.9%	Z=1.44 (p=.15)

n = Number of studies, N = Number of subjects, Ne = Number of subjects in experimental group; Nc = Number of subjects in control group.

A composite moderate and statistically significant effect size (Cohen’s *d*) was observed for subjective well-being d = 0.34 (95% CI [0.22, 0.45], p<.01). For psychological well-being, Cohen’s *d* was 0.20 (95% CI [0.09, 0.30], p<.01) and for depression d = 0.23 (95% CI [0.09, 0.38], p<.01), which can be considered as small.

Heterogeneity was moderate for subjective well-being (I^2^ = 49.5%) and depression (I^2^ = 47.0%), and low for psychological well-being (I^2^ = 29.0%). Effect sizes ranged from −0.09 [66] to 1.30 [64] for subjective well-being, -0.06 [78] to 2.4 [83] for psychological well-being and −0.17 [69] to 1.75 [83] for depression.

Removing outliers reduced effect sizes for all three outcomes: 0.26 (95% CI [0.18, 0.33], Z=6.43, p<.01) for subjective well-being (Burton & King, 2004 and Peters et al., 2010 removed) [64,70], 0.17 (95% CI [0.09, 0.25], Z=4.18, p<.01) for psychological well-being (Fava et al. (2005) removed) [83] and 0.18 (95% CI [0.07, 0.28], Z=3.33, p<.01) for depression (Fava, 2005 and Seligman, 2006 study 2, removed) [51,83]. Removing the outliers reduced heterogeneity substantially (to a non-significant level).

### Follow-up effects

Ten studies examined follow-up effects after at least three months and up to 12 months (Table 3). For the purposes of interpretation, we used only those studies examining effects from three to six months (short-term follow-up), thus excluding Fava et al. (2005) [83] which had a follow-up at one year. The random-effects model demonstrated small but significant effects in comparison with the control groups for subjective well-being (Cohen’s *d* 0.22, 95% CI [0.05, 0.38], p<.01) and for psychological well-being (0.16, 95% CI [0.02, 0.30], p = .03). The effect was not significant for depression (0.17, 95% CI [−0.06, 0.39], p = .15). Heterogeneity was low for subjective well-being (I^2^ = 1.1%) and psychological well-being (I^2^ = 26.0%), and high for depression (I^2^ = 63.9%).

### Subgroup analyses

Subgroup analyses are presented in Table 4. We looked at self-selection, duration of the intervention, type of intervention, recruitment method, application of inclusion criteria related to certain psychosocial problems, and quality rating.

**Table 4:** Moderator effects: subgroup analysis (post-test)

**Outcome measure**	**Criteria**	**Subgroup (study)**	**n**	**Cohen’s d (95% CI)**	**Test for subgroup differences**
Subjective well-being	Self-selection	Self-selected	15	0.29 (0.18 – 0.39)***	Q=0.64, df=1 (p=.43)
		Not self-selected	13	0.38 (0.17 – 0.60)**	
	Duration	<=4 weeks	17	0.35 (0.18 – 0.52)***	Q=1.84, df=2 (p=.91)
		<=8 weeks	6	0.24 (0.10 - 0.39)**	Slope=0.01, Z=0.14 (p=.89)
		>8 weeks	5	0.43 (0.17 – 0.68)**	
	Type	Self help	20	0.33 (0.20 – 0.46)***	Q=0.20, df=2 (p=.91)
		Group	5	0.38 (0.03 – 0.73)*	
		Individual	3	0.41 (0.01 – 0.81)*	
	Recruitment	Community	6	0.29 (0.11 – 0.48)**	Q=5.36, df=4 (p=.25)
		Internet	2	0.06 (−0.24 – 0.35)ns	
		Referral/hospital	2	0.51 (0.08 – 0.95)*	
		University	16	0.36 (0.19 – 0.53)***	
		Organization	2	0.62 (0.11-1.12)*	
	Psychosocial problems	Specific psychosocial problems	4	0.31 (0.09 – 0.52)**	Q=0.10, df=1 (p=.76)
		None	24	0.35 (0.22 – 0.48)***	
	Quality rating	Low (1–2)	16	0.29 (0.17 – 0.40)***	Q=2.41, df=2 (p=.30)
		Medium (3–4)	11	0.40 (0.19 – 0.61)***	Slope=−0.00, Z=0.08 (p=.94)
		High (5–6)	1	0.05 (−0.34 – 0.44)ns	
Psychological well-being	Self-selection	Self-selected	15	0.18 (0.05 – 0.30)**	Q=0.32, df=1 (P=.57)
		Not self-selected	5	0.25 (0.04- 0.46) *	
	Duration	<=4 weeks	11	0.16 (0.07 – 0.25)***	Q=1.91, df=2 (p=.39)
		<=8 weeks	2	0.35 (−0.20 – 0.89)ns	Slope=0.05, Z=0.95 (p=.34)
		>8 weeks	7	0.41 (0.03 – 0.79)*	
	Type	Self help	10	0.14 (0.05 – 0.23)**	Q=3.76, df=2 (p=.15)
		Group	6	0.26 (0.08 – 0.44)**	
		Individual	4	0.81 (−0.01 – 1.63)ns	
	Recruitment	Community	4	0.20 (−0.03 – 0.44)ns	Q=7.04, df=4 (p=.13)
		Internet	5	0.09 (−0.03 – 0.21)ns	
		Referral/hospital	4	0.91 (0.24 – 1.57)**	
		University	5	0.22 (0.08 – 0.35)**	
		Organization	2	0.18 (−0.43 – 0.78)ns	
	Psychosocial problems	Specific psychosocial problems	5	0.59 (0.00 – 1.18)*	Q=1.93, df=1 (p=.17)
		None	15	0.17 (0.08 – 0.25)***	
	Quality rating	Low (1–2)	10	0.32 (0.07 – 0.58)*	Q=1.86, df=2 (p=.40)
		Medium (3–4)	9	0.15 (0.06 – 0.24)**	Slope=−0.01, Z=−0.45 (p=.66)
		High (5–6)	1	0.29 (−0.11 – 0.68)ns	
Depression	Self-selection	Self-selected	12	0.20 (0.05– 0.36)*	Q=1.73, df=1 (p=.19)
		Not self-selected	2	0.41 (0.15 – 0.66)**	
	Duration	<=4 weeks	7	0.15 (0.02 - 0.28)*	Q=4.86, df=2 (p=.09)
		<=8 weeks	2	0.47 (−0.02 - 0.97)ns	Slope=0.20, Z=2.32 (p=.02)
		>8 weeks	5	0.68 (0.15 – 1.21)*	
	Type	Self help	8	0.15 (0.03 – 0.27)*	Q=6.99, df=2 (p=.03)
		Group	2	0.47 (−0.02 – 0.97)ns	
		Individual	4	0.88 (0.29 – 1.47)**	
	Recruitment	Community	1	0.48 (−0.23 – 1.18)ns	Q=15.76, df=4 (p<.01)
		Internet	5	0.11 (−0.02 – 0.23)ns	
		Referral/hospital	3	1.14 (0.55 – 1.73)***	
		University	3	0.41 (0.17 – 0.65)**	
		Organization	2	0.22 (−0.18 – 0.63)ns	
	Psychosocial problems	Specific psychosocial problems	5	0.78 (0.35 – 1.21)***	Q=7.65, df=1 (p=.01)
		None	9	0.16 (0.05 – 0.27)**	
	Quality rating	Low (1–2)	7	0.47 (0.26 – 0.67)***	Q=10.14, df=2 (p=.01)
		Medium (3–4)	6	0.15 (0.00 – 0.30)*	Slope=−0.10, Z=−2.26 (p=.02)
		High (5–6)	1	−0.17 (−0.56 – 0.22)ns	

* p<0.05; ** p<0.01; *** p<0.001; ns non-significant.

n = Number of studies.

For depression, five out of six subgroups of studies resulted in significantly higher effect sizes. Higher effect sizes were found for 1) interventions of a longer duration (only in the meta regression analysis), 2) individual interventions, 3) studies involving referral from a health care practitioner or hospital, 4) studies which applied inclusion criteria based on psychosocial problems and 5) lower quality studies. For subjective well-being and psychological well-being, there were no significant differences between subgroups, although for the latter there was a recognizable trend in the same direction and on the same moderators, except for quality rating.

Twenty-six out of 39 studies were self-help interventions for which we conducted a separate subgroup analysis. However, there was little diversity within the self-help subgroup: only six studies examined intensive self-help for longer than four weeks, self-help was offered to people with specific psychosocial problems in only one study and more than half of the self-help studies (n=14) recruited their participants via university. Consequently, there were no significant differences between subgroups for self-help interventions.

### Publication bias

Indications for publication bias were found for all outcome measures, but to a lesser extent for subjective well-being. Funnel plots were asymmetrically distributed in such a way that the smaller studies often showed the more positive results (in other words, there is a certain lack of small insignificant studies). Orwin’s fail-safe numbers based on a criterium effect size of 0.10 for subjective well-being (59), psychological well-being (16) and depression (13) were lower than required (respectively 150, 110 and 80). Egger’s regression intercept also suggests that publication bias exists for psychological well-being (intercept=1.18, t=2.26, df=18, p=.04) and depression (intercept=1.45, t=2.26, df=12, p=.03), but not for subjective well-being (intercept=1.20, t=1.55, df=26, p=0.13). The mean effect sizes of psychological well-being and depression were therefore recalculated by imputing missing studies using the Trim and Fill method. For psychological well-being, three studies were imputed and the effect size was adjusted to 0.16 (95% CI 0.03-0.29). For depression, five studies were imputed and the adjusted effect size was 0.16 (95% CI 0.00-0.32).

## Discussion

### Main findings

This meta-analysis synthesized effectiveness studies on positive psychology interventions. Following a systematic literature search, 40 articles describing 39 studies were included. Results showed that positive psychology interventions significantly enhance subjective and psychological well-being and reduce depressive symptoms. Effect sizes were in the small to moderate range. The mean effect size on subjective well-being was 0.34, 0.20 on psychological well-being, and 0.23 on depression. Effect sizes varied a great deal between studies, ranging from below 0 (indicating a negative effect) to 2.4 (indicating a very large effect). Moreover, at follow-up from three to six months, small but still significant effects were found for subjective well-being and psychological well-being, indicating that effects were partly sustained over time. These follow-up results should be treated with caution because of the small number of studies and the high attrition rates at follow-up.

Remarkably, effect sizes in the current meta-analysis are around 0.3 points lower than the effect sizes in the meta-analysis by Sin and Lyubomirsky (2009) [37]. We included a different set of studies in which the design quality was assured using randomized controlled trials only. Effectiveness research in psychotherapy shows that effect sizes are relatively small in high-quality studies compared with low-quality studies [35] and this might also be true for positive psychology interventions. In addition, we applied stricter inclusion criteria than those used by Sin and Lyubomirsky (2009) and therefore did not include studies on any related areas such as mindfulness and life review therapy. These types of interventions stem from long-standing independent research traditions for which effectiveness has already been established in several meta-analyses [40,41]. Also, the most recent studies were included. This might explain the overestimation of effect sizes in the meta-analysis by Sin and Lyubomirsky (2009).

Several characteristics of the study moderated the effect on depressive symptoms. Larger effects were found in interventions with a longer duration, in individual interventions (compared with self-help), when the interventions were offered to people with certain psychosocial problems and when recruitment was carried out via referral from a health care professional or hospital. Quality rating also moderated the effect on depression: the higher the quality, the smaller the effect. Interestingly, these characteristics did not significantly moderate subjective well-being and psychological well-being. However, there was a trend in the moderation of psychological well-being that was the same as that observed in the studies which included depression as an outcome. In general, effectiveness was increased when interventions were offered over a longer period, face-to-face on an individual basis in people experiencing psychosocial problems and when participants were recruited via the health care system.

Although it is clear that more intensive and face-to-face interventions generate larger effects, the effects of short-term self-help interventions are small but significant. From a public health perspective, self-help interventions can serve as cost-effective mental health promotion tools to reach large target groups which may not otherwise be reached [86-88]. Even interventions presenting small effect sizes can in theory have a major impact on populations’ well-being when many people are reached [89]. The majority of positive psychology interventions (in our study 26 out of 39 studies) are already delivered in a self-help format, sometimes in conjunction with face-to-face instruction and support. Apparently, self-help suits the goals of positive psychology very well and it would be very interesting to learn more about how to improve the effectiveness of PPI self-help interventions. However, a separate subgroup analysis on the self-help subgroup revealed no significant differences in the present meta-analysis. There was very little variation in the subgroups as regards population, duration of the intervention and recruitment method. As a result, this analysis does not give firm indications on how to improve the effectiveness of self-help interventions. It is possible that self-help could be enhanced by offering interventions to people with specific psychosocial problems, increasing the intensity of the intervention and embedding the interventions in the health care system. However, more studies in diverse populations, settings and with varying intensity are needed before we can begin to derive recommendations from this type of meta-analysis. Other research gives several additional indications on how to boost the efficacy of self-help interventions. Adherence tends to be quite low in self-help interventions [90,91] and therefore, enhancing adherence could be a major factor in improving effectiveness. Self-help often takes a ‘one size fits all’ approach, which may not be appropriate for a large group of people who will, as a consequence, not fully adhere to the intervention. Personalization and tailoring self-help interventions to individual needs [92] as well interactive support [93] might contribute to increased adherence and likewise improved effectiveness of (internet) self-help interventions.

### Study limitations

This study has several limitations. First, the quality of the studies was not high, and no study met all of our quality criteria. For example, the randomization procedure was unclear in many studies. Also, most studies conducted completers-only analysis, as opposed to intention-to-treat analysis. This could have seriously biased the results [35]. However, the low quality of the studies could have been overstated as the criteria were scored conservatively: we gave a negative score when a criterion was not reported. Even so, more high-quality randomized-controlled trials are needed to enable more robust conclusions about the effects of PPIs. Second, different types of interventions are lumped together as positive psychology interventions, despite the strict inclusion criteria we applied. As expected, we found a rather high level of heterogeneity. In the future, it might be wise and meaningful to conduct meta-analyses that are restricted to specific types of interventions, for example gratitude interventions, strengths-based interventions and well-being therapy, just as has already been carried out with, for example, mindfulness and life review. In the present meta-analysis, studies on these specific interventions were too small and too diverse to allow for a subgroup-analysis. Third, the exclusion of non peer-reviewed articles and grey literature could have led to bias, and possibly also to the publication bias we found in our study. Fourth, although we included a relatively large number of studies in the meta-analysis, the number of studies in some subgroups was still small. Again, more randomized-controlled trials are needed to draw firmer conclusions. Sixth, the study of positive education is an emerging field in positive psychology [94-98] but school-based interventions were excluded from our meta-analysis due to the strict application of the inclusion criteria (only studies with randomization at individual level were included).

## Conclusion

This meta-analysis demonstrates that positive psychology interventions can be effective in the enhancement of subjective and psychological well-being and may help to reduce depressive symptom levels. Results indicate that the effects are partly sustained at short-term follow-up. Although effect sizes are smaller in our meta-analysis, these results can be seen as a confirmation of the earlier meta-analysis by Sin and Lyubomirsky (2009). Interpretation of our findings should take account of the limitations discussed above and the indications for publication bias.

### Implications for practice

In mental health care PPIs can be used in conjunction with problem-based preventive interventions and treatment. This combination of interventions might be appropriate when clients are in remission; positive psychology interventions may then be used to strengthen psychological and social recourses, build up resilience and prepare for normal life again. On the basis of the moderator analysis, we would recommend the delivery of interventions over a longer period (at least four weeks and preferably eight weeks or longer) and on an individual basis. Practitioners can tailor their treatment strategy to the needs and preferences of a client and can use positive psychology exercises in combination with other evidence-based interventions that have a positive approach and aim to enhance well-being, such as mindfulness interventions [40], Acceptance and Commitment Therapy [7,99], forgiveness interventions [42], behavioral-activation [100] and reminiscence [41,101].

In the context of public health, positive psychology interventions can be used as preventive, easily accessible and non-stigmatizing tools. They can potentially be used in two ways: 1) in mental health promotion (e.g. leaflets distributed for free at community centers, (mental) health internet portals containing psycho-education), and 2) as a first step in a stepped care approach. In the stepped care model, clients start with a low-intensity intervention if possible, preferably a self-directed intervention. These interventions can be either guided by a professional or unguided, and are increasingly delivered over the internet. Clinical outcomes can be monitored and people can be provided with more intensive forms of treatment, or referred to specialized care, if the first-step intervention does not result in the desired outcome [102].

### Recommendations for research

Regarding the research agenda, there is a need for more high-quality studies, and more studies in diverse (clinical) populations and diverse intervention formats to know what works for whom. Standards for reporting studies should also be given more attention, for example by reporting randomized controlled trials according to the CONSORT statement [103]. In addition, we encourage researchers to publish in peer-reviewed journals, even when the sample sizes are small or when there is a null finding of no effect, as this is likely to reduce the publication bias in positive psychology. Furthermore, most studies are conducted in North America. Therefore, replications are needed in other countries and cultures because some positive psychology concepts may require adaptation to other cultures and outlooks (e.g. see Martinez et al., 2010) [68]. Last but not least, we strongly recommend conducting cost-effectiveness studies aiming to establish the societal and public health impact of positive psychology interventions. This type of information is likely to help policy makers decide whether positive psychology interventions offer good value for money and should therefore be placed on the mental health agenda for the 21^st^ century.

## Competing interests

The authors declare that they have no competing interests.

## Authors’ contributions

LB conducted the meta-analysis, including the literature selection and data-analysis, and wrote the manuscript. MH took care of selecting the articles and cross-checking the data. All authors contributed to the design of the study. EB, GW, HR and FS are advisors in the project. All authors provided comments and approved the final manuscript.

## Pre-publication history

The pre-publication history for this paper can be accessed here:

http://www.biomedcentral.com/1471-2458/13/119/prepub
